# Fungal Metagenome of Chernevaya Taiga Soils: Taxonomic Composition, Differential Abundance and Factors Related to Plant Gigantism

**DOI:** 10.3390/jof7110908

**Published:** 2021-10-27

**Authors:** Mikhail Rayko, Sophie Sokornova, Alla Lapidus

**Affiliations:** 1 Center for Algorithmic Biotechnology, St. Petersburg State University, 199004 Saint-Petersburg, Russia; a.lapidus@spbu.ru; 2 Department of Phytotoxicology and Biotechnology, All-Russian Institute of Plant Protection, 196608 Saint-Petersburg, Russia; svsokornova@vizr.spb.ru

**Keywords:** fungi, diversity, community composition, plant gigantism, arbuscular mycorrhiza, Chernevaya taiga, soil properties, FUNGuild

## Abstract

The Chernevaya taiga of Western Siberia is a unique and complex ecosystem, distinguished by the unusually large sizes of herbaceous plants, the reasons for which are poorly understood. Here, we explored the fungal diversity of the Chernevaya taiga soils in the Tomsk regions of Western Siberia in comparison with other soil types. The soil biomes of Chernevaya taiga and the control regions were investigated using Illumina ITS rRNA sequencing, and taxonomic analysis revealed a predominance of fungal phyla in the different soils. These results demonstrate that the fungi of the Chernevaya taiga regions have a higher species diversity (Faith’s PD) vs. the control soils, and the diversity is due more to the sampling sites rather than to the seasons (Bray-Curtis distance). We studied most of the differentially abundant taxa among the soil types, and we annotated the taxa with their ecological guilds and trophic types. Some of the abundant fungal taxa in the summer- and fall-Chernevaya taiga samples belong to the phylum Glomeromycota—arbuscular mycorrhizal symbiotrophs, which are known to establish symbiotic relationships and enhance plant growth. Additionally, several OTUs were assigned to novel genera in the Glomeraceae and Claroideoglomeraceae families. Our findings add a potential explanation of the high productivity and plant gigantism in Chernevaya taiga and expand our knowledge of fungal biodiversity.

## 1. Introduction

The forests of Chernevaya taiga represent unique boreal forest formations that are limited in their distribution by hyper-humid sectors of the Altai-Sayan mountain region. These ecosystems are characterized by several features: (i) the growth of two main dominant tree species—Siberian fir (*Abies sibirica*) and common aspen (*Populus tremula*); (ii) tall-growing shrubs such as Siberian mountain-ash (*Sorbus sibirica*), birds cherry (*Padus avium*), and goat willow (*Salix caprea*) that can reach up to 18 m in height and 30 cm in diameter; (iii) dense layers of large perennial herbs of up to 4.5 m; (iv) several species representative of Tertiary nemoral relict flora; (v) an absence of moss cover on the soil surface [1].

The soils of Chernevaya taiga represent some of the most interesting but not yet studied subjects of metagenomic research, since they demonstrate several unusual features, such as extremely tall grasses with an overall low concentration of humus (effective fertility), and an unusually high decomposition rate of plant residues. Obviously, both the productivity of Chernevaya taiga soils and the rapid decomposition of leaf litter are a complex of related properties, the analysis of which in the framework of a metagenomic study is an important fundamental task [2]. In addition, the study of these soils can be very important from a practical point of view, as they are sources of cellulolytic strains, antibiotics and a variety of other biologically active compounds that are of fundamental and practical importance.

The role of fungi in the decomposition of plant litter and carbon cycling [3,4], and in the plant vegetation properties [5], is well-known. Different types of mycorrhizal-forming fungi allow the host plants to obtain nutrients from the soil, including phosphate, and diffusion-limited elements such as Zn and Cu [6]. In turn, host plants provide fungi with sugars, thereby forming symbiotic relationships. The hyphae of the mycorrhizal fungi, which spread beyond the nutrient depletion area, naturally extend the plant root system and allow access to nutrients that would be otherwise unavailable. Another important role of the mycorrhiza is to increase water availability by increasing the suction surface area. Additionally, symbiotic fungi may play a protective role by working as barriers to pathogens, providing the antibiotic compounds and accumulating heavy metals from the soil [7].

One of the most striking examples is the arbuscular mycorrhizal (AM) fungi of the phylum Glomeromycota. Forming intraradical hyphae, the AM increases the uptake of nutrients and facilitates the transfer of phosphorus and nitrogen to the plant under limiting conditions [8,9]. This highly evolved symbiosis was established in the early ancestors of land plants, and likely played a role in land colonization [10,11]. It was shown that the AM fungi and beneficial saprophytic mycoflora are capable of promoting plant growth [12], and there is a relationship between the AM and the synthesis of phytohormones [13].

Along with the AM, it was shown [14] that boreal forests are heavily dependent on ectomycorrhizal associates—towards the north borders, where the harsh conditions limit the proliferation of the ectomycorrhizal fungi, forests show lower productivity. In the case of Chernevaya taiga, the region with high humidity, ectomycorrhizal fungi can serve as a productivity-driver for the whole ecosystem.

Until recently, Chernevaya taiga soils were studied very superficially, the phenomenon of plant gigantism was described only morphologically, and just a few attempts were made to find the cause of this peculiarity, mostly in terms of environmental and climatic factors. At the same time, the identification of such features could have an impact on agricultural technology.

One of the reasons could be geographical features, such as high humidity and peculiarities of snow cover, but the results of vegetation experiments show that when the soil is extracted and used for growing control plants in lab conditions, the effect of increased productivity is maintained; therefore, the microbial components of the soil, such as bacteria and fungi, could be the drivers of fertility.

In this study we explored the composition and properties of the Chernevaya taiga fungal communities, and their roles in the unique features of this ecosystem.

## 2. Materials and Methods

### 2.1. Soil Sampling

In May 2020, field expeditions were conducted to collect samples of model plant species and soil samples at key sites of the Chernevaya taiga of Salair, and in the grass pine forest on the ancient terraces of the Ob River (see detailed coordinates in Table 1). At each sampling point, five soil samples were obtained as biological replicates.

### 2.2. DNA Extraction and Sequencing

DNA from the 30 soil samples was extracted according to the protocol currently adopted in the Earth Microbiome Project [15]. Fungal DNA was amplified according to the EMP ITS Illumina amplification protocol [16]. Libraries were sequenced on the Illumina MiSeq instrument in the paired-end mode. Extraction, purification and sequencing of DNA were performed in the Institute of Cytology and Genetics, Novosibirsk, as part of the large initiative on study of the Chernevaya taiga soils.

### 2.3. Plant Sample Preparation for Microscopy Visualization

Plant material fixed in Carnoy’s solution was used to make thick sections (40) by cryo-microtome. The visualization of the AM fungal structures as bright-field images using wheat germ agglutinin (WGA) coupled to 3,3′-diaminobenzidine (DAB) was carried out according to [17].

### 2.4. Statistical and Bioinformatics Analysis

Analysis was performed using QIIME2 [18] and additional R scripts, with the following steps: a. Demultiplexing, QC, adapter trimming. On this step the adapters and barcodes were removed, sequencing reads were associated with corresponding samples. b. Denoising, amplicon sequence variant selection. DADA2 approach was used to correct amplicon sequence data and obtain a table of amplicon sequence variants (ASVs), which are higher-resolution analogues of the traditional OTUs, providing an abundance of each ASV in each sample.

c. Core diversity metrics calculation. Alpha-diversity (diversity within an ecosystem) was calculated using Faith’s PD index, beta-diversity—using UniFrac metric. d. Taxonomy assignment according to UNITE database v. 8.2 [19]. Pre-trained classifier for QIIME2 format was used to assign certain taxonomic rank to each ASV.

e. Differential abundance was calculated using phyloseq [20] and Deseq2 [21] packages. Pairwise comparison of samples from Chernevaya taiga and control samples from each season was performed in order to detect ASVs that were significantly more abundant in certain samples. The threshold for the log2 fold change (l2FC) was set at 2.0 and the FDR-adjusted p-value was cutoff at 0.05

Association of the fungal taxa with ecological guilds was performed using the FUNGuild approach [22]. All the commands and parameters used in QIIME2 analysis steps are contained in provenance section of the QIIME2 artifact files openly available in FigShare at https://doi.org/10.6084/m9.figshare.16565721.v3 (accessed on 12 September 2021).

## 3. Results

In order to study the relationships between Chernevaya taiga fungi and plant gigantism, we explored the diversity of the fungal communities, taxonomic composition and relative abundance between the Chernevaya taiga and control samples. A higher biodiversity usually corresponds to a higher soil quality and nutrient supply, and can serve as a soil health indicator [5]. The taxonomic composition reveals the most common taxa in different samples, and the relative abundance reveals those taxa that contribute to the characteristics of particular soils.

### 3.1. Diversity

**Figure 1:**
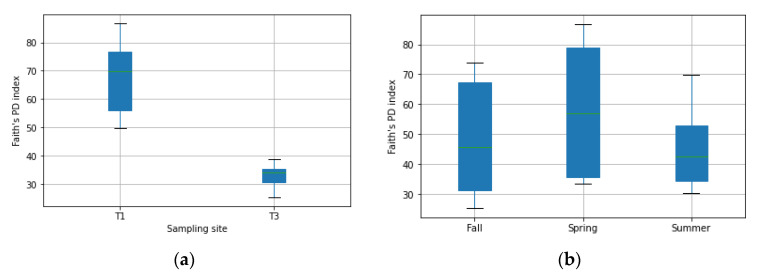
Alpha diversity (species richness) in the upper soil layer. (**a**)difference in sampling sites, (**b**)difference between collection seasons. Faith’s PD index reflects the diversity of ASVs in samples—the greater the Faith’s PD index, the higher the diversity of the microbiota. Boxplots represent the interquartile range (IQR) between the 1st and 3rd quartiles, and the horizontal line defines the median.

We performed the diversity analysis in order to study the fungal community structures and the impact of the soil type and season. First, we analyzed the alpha-diversity, which is a measure of the species richness of a particular sample, using the Faith’s phylogenetic diversity index (Faith’s PD). The results of the alpha-diversity analysis show that the Chernevaya taiga samples have significantly higher diversity, and that the time of the year does not have a significant impact (see Figure 1). At the same time, it is shown that there is usually an observed seasonal fluctuation of fungal diversity in cool-temperate forest communities [23].

**Figure 2:**
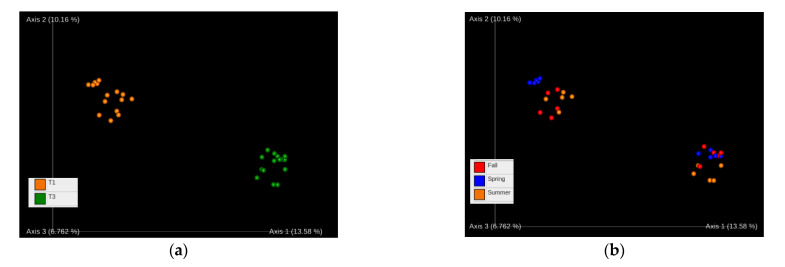
Analysis of β-diversity: weighted UniFrac [24], topsoil. (**a**) Samples according to collection point (T1, T3); (**b**) Samples according to season (Fall 2019, Spring 2020, Summer 2020).

The beta-diversity analysis was performed in order to compare the fungal communities from the different sampling sites. We used a weighted UniFrac distance metric [24] that showed the dissimilarity between communities, based on species content and phylogenetic information. Our results show the clear separation of the Chernevaya taiga samples from the control samples by their fungal community compositions. Again, the species composition was consistent between seasons, which demonstrates the sustainability of the fungal communities in the studied soils (Figure 2).

### 3.2. Community Composition

**Figure 3:**
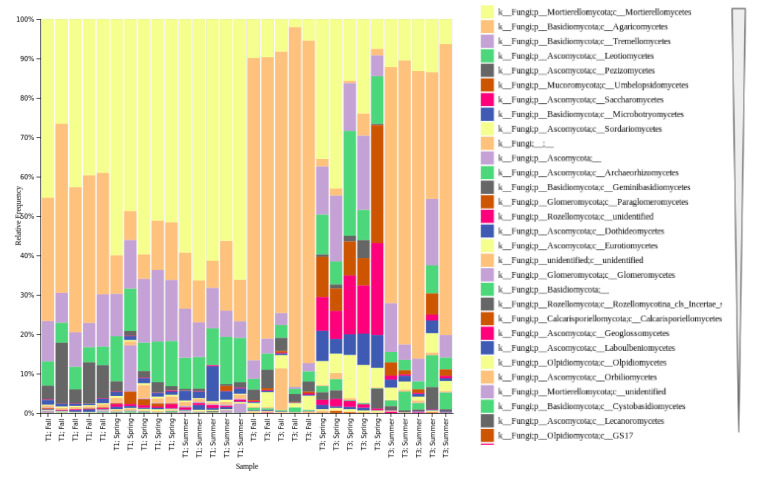
Most abundant fungal classes in soil samples based on ITS analysis, separated by sampling site and season (T1—Chernevaya taiga, T3—control soils). UNITE database v.8.2 [19] was used for the taxonomic assignment.

We analyzed the fungal taxonomic composition of the soil samples in each season and sampling site. When the results were grouped at the class level, the most abundant class of fungi in the Chernevaya taiga samples belonged to the Mortierellomycetes, followed by the Agaricomycetes (Figure 3). After breaking down these results to the species level, all of the Mortierellomycetes abundance was comprised of only 4 *Mortierella* species, which demonstrates the domination of a single genus across all of the fungal communities. It was followed by yeast *Solicoccozyma* (Tremellomycetes) and mushroom-forming *Inocybe* (Agaricomycetes) species, which is typical for boreal forests [25].

We observed a significant abundance of Agaricomycetes in the summer and fall seasons in the control samples. In the Chernevaya taiga soils, we also saw an increase in the number of Agaricomycetes in the fall (albeit in much lower amounts than in the control samples), which reflects their seasonal dynamics. Although Agaricomycetes are symbiotrophs, their main role in the ecosystem is the lignification of woody debris [26]. Thus, it is not surprising to observe a high amount of such fungi in forest soils. The notable feature of Chernevaya taiga is the enormous size of the grasses and shrubs, rather than the woody plants, and there are a significant number of saprotrophs that play a role in the decomposition of leaf litter.

### 3.3. Ecological Guild Analysis

**Figure 4:**
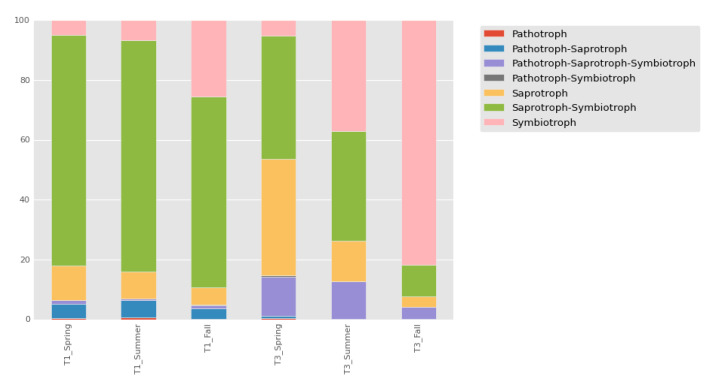
Relative abundance of fungi with various trophic modes in the different sampling sites (T1—Chernevaya taiga, T3—control soils).

As a next step, we studied the global picture of the ecological properties of the communities using the FUNGuild approach [20]. The ratio of the different trophic modes in the samples from Chernevaya taiga remained more stable at different seasons (see Figure 4). We observed a dominance of saprotrophs and symbiotrophs in the Chernevaya taiga samples. It was expected to see an abundance of saprotrophs, given the high decomposition rate of the leaf litter in this ecosystem, but the significant amount of symbiotrophs requires a more thoughtful examination.

Given the prevalence of the saprotrophs and symbiotrophs in the Chernevaya taiga samples, we examined the species content in more detail. Based on the normalized read count, the vast majority (75.2%) of saprotrophs and symbiotrophs belong to different *Mortierella* species. The species of *Mortierella* live as saprotrophs in the soil, playing an important role in the decaying of leaf litter and other organic materials, and can likely impact the soil fertility, as shown previously [27,28]. This result is consistent with the community composition analysis, which suggests *Mortierella* as one of the major fertility drivers in the Chernevaya taiga soils.

### 3.4. Differentially Abundant Fungal Taxa

Along with the detection of the absolute abundance of *Mortierella* in the soil samples, we have analyzed the taxa that are differentially abundant among the soil types, in order to investigate the less-abundant fungi that, nevertheless, may play an important role in the soil fertility (Figure S1). The ASVs were grouped on the phylum level, and for each phylum we calculated the log2 fold change (l2FC). The results are shown in Table 2.

On the phylum level, we observed an increase in Mortierellomycota, Rozellomycota and Glomeromycota in the Chernevaya taiga soils. Again, all of the Mortierellomycota were represented by just a few species of *Mortierella* saprotrophs.

Rozellomycota, which are parasitic microorganisms, differ from classical fungi per se, and are represented in our data by three orders of Rozellomycotina *incertae sedis* (unidentified genus of GS03, GS05 and GS08 order). Unlikely to establish symbiosis with plants, they nevertheless can grow on substrates containing chitin (insects, fungi), cellulose (fallen leaves) and keratin (feathers, etc.), and we may assume that they can also take part in leaf-litter decomposition.

The ecosystem and litter type, in general, may have a significant influence on composition of the fungal species in the soil [29]. Rozellomycota have been shown to become the dominant species in the late stages of the decomposition of wetlands [30].

The most differentially abundant taxa in the summer and fall samples (log2 fold change −4.11 between Chernevaya taiga and control soils) belong to the phylum Glomeromycota—arbuscular mycorrhizal symbiotrophs, which are known to establish symbiotic relationships and enhance plant growth [8].

### 3.5. Arbuscular Mycorrhiza in the Chernevaya Taiga Soils

**Figure 5:**
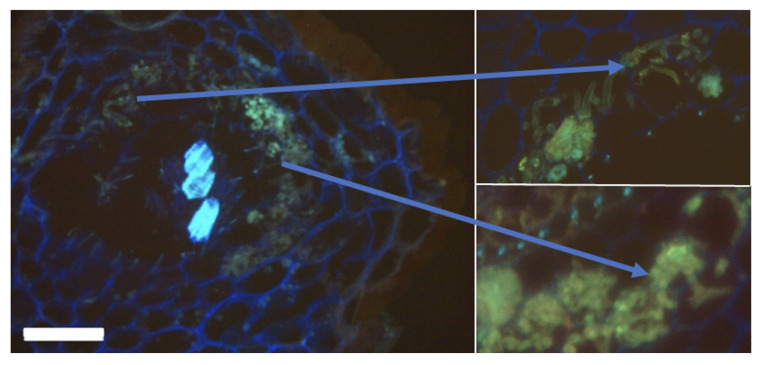
*Aconitum septentrionale* root samples colonized by arbuscular mycorrhiza fungi. Cryomicroscopy section, thickness 40 µm. Fungal walls are stained with DAPI-WGA (green). Bars 100 μm.

Some species of herbs in the Chernevaya Taiga may reach exceptional sizes. In our study, *Aconitum septentrionale* and *Crepis sibirica* were chosen as model herbs for this ecosystem. *A. septentrionale*, an herbaceous perennial plant, does not usually form an association with arbuscular mycorrhiza (to the best of our knowledge). We took three root samples from each set of samples that were collected in the summer of 2019 at locations T1 and T3, respectively. From these, we prepared slices for cryomicroscopic imaging (see Methods section for details). Only in the Chernevaya taiga samples did the roots of *A. septentrionale* demonstrate the presence of arbuscular mycorrhizal fungi (see Figure 5). We believe that the unique ecological conditions of this ecosystem contribute to the formation of symbiotic relationships of plants with fungi.

Moreover, new symbiotic taxa have been identified—several OTUs were assigned to novel genera in the Glomeraceae and Claroideoglomeraceae families, which are absent in the UNITE fungal ITS database. Table 3 shows the ASVs that correspond to the unidentified taxa within Glomeromycota, which are differentially abundant among the soil types studied. These potential novel genera deserve further study because arbuscular mycorrhizal fungi usually cannot be cultivated.

**Figure 6:**
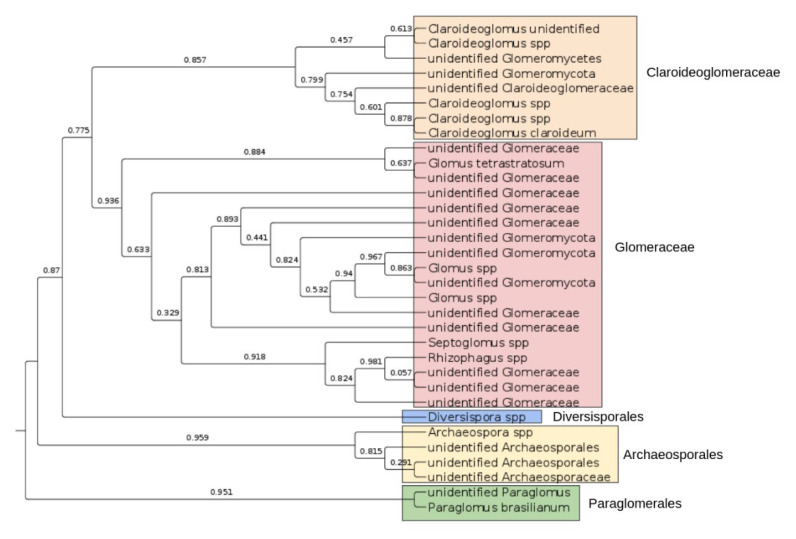
Phylogenetic tree of the Glomeromycota species. Sequences are clustered at 90% similarity, and cluster representatives are aligned with MAFFT. Tree was inferred with maximum likelihood approach in FastTree software, node support obtained by SH test with 1000 resamples.

We reconstructed the phylogenetic tree of all Glomeromycota taxa that were found in our study (Figure 6). Similar amplicon sequence variants (ASVs) were clustered at the 90% similarity level, in order to depict the phylogenetic structures of the Glomeromycota in the soil samples. We observed the presence of all four orders of Glomeromycota—Paraglomerales, Archaeosporales, Diversisporales and Glomerales. The vast majority of the novel species and genera correspond to the Glomerales order, which contain both Glomeraceae and Claroideoglomeraceae families. The observed phylogenetic diversity of Glomeromycota species is typical for a boreal, herb-rich and coniferous forest [31].

## 4. Discussion

The productivity of boreal forest soils is largely impacted by fungal composition. The ecosystem in question, Chernevaya taiga of Western Siberia, is not an exception. Chernevaya taiga is an unusual ecosystem compared to other boreal forests. In this study, we attempted to establish the specific nature of its fungal component, which shapes this ecosystem and contributes to its unusual level of productivity.

According to vegetation experiments [32], the fertility of the Chernevaya taiga soils remains high after extraction and shows an increase in plant growth in lab conditions, with the same humidity and temperature, compared to the control soil samples. That observation suggests that the vegetation impact is determined by the soil itself rather than the climate, and that the fertility driver (some part of the fungal or microbial component, or both) can be potentially determined and used in agriculture technologies.

According to Lindahl and Clemmensen [14], in typical boreal conifer forests, the ecosystem production is generally constrained by low nitrogen availability, and durable litter with a low nutrient content hampers decomposition. Nevertheless, the relative fungal/plant diversity in boreal forests is quite high [33]. Study of the physico-chemical properties of the Chernevaya taiga soils showed an average or high nitrogen content (in ammonium and nitrate forms in upper horizons), which is at least partially explained by the high number of herbaceous remnants (whose nitrogen content is much higher than woody remnants) [2].

The predominance of the nitrate forms of nitrogen, which are easily accessible to plants (especially in the upper horizons), indicates the important role of nutrient intake from the litter. Plant litter decomposition is a key step in nutrient recycling [34] and leads to nitrogen abundance. An excess of nitrogen reduces the competition for substrates between symbiotic fungi and bacteria, which can lead to a unique and extremely favorable situation for plant development. In this regard, special attention should be paid to fungi that act as litter decomposers.

Based on the results obtained in the current work, the fungi of the Chernevaya taiga regions have a higher species diversity, which is determined by the sampling sites rather than the seasons. This observation is consistent with the data that boreal-forest fungal communities are usually clustered by site, thereby maintaining the vertical pattern of fungi segregation in different horizons [35].

Our analysis of the taxonomic composition of the soil samples in different seasons and sampling sites revealed the Mortierellomycota, Basidiomycota and Ascomycota phyla as the most abundant taxa. It is assumed that Ascomycota members predominantly participate in the early stages of leaf-litter decomposition, then are displaced by the Basidiomycota phylum, especially basidiomycetous yeasts [36]. In general, a similar trend was observed for both boreal and non-boreal taiga. At the same time the most abundant class of fungi in the Chernevaya taiga samples belonged to Mortierellomycetes—saprotrophs found in soil, on decaying leaves and other organic material. Just a few species account for almost half of the genetic material of fungi in the soils of the samples. As shown in [28], the *Mortierella* species may serve as very valuable decomposers, increasing the nutrient uptake efficiency, utilizing carbon sources (including polymers such as cellulose or chitin) and promoting plant growth.

Among the most differentially abundant taxa, we also found members of the phylum Glomeromycota—arbuscular mycorrhizal symbiotrophs. It was shown that they can increase nutrient and water availability, hormonal regulation, and the mycorrhiza-induced resistance of host plants [8,9]. Arbuscular mycorrhizal fungi can create a network of hyphae that connects the roots of many plants. These networks allow nutrients to be redistributed between plants, resulting in some plants growing better, and others worse [37]. In the unique conditions of boreal forests, we believe that nutrient surpluses lead to the giant growth of some plants and the normal development of others, because of the nutrient availability. Additionally, it was shown that colonization of the AM fungi can lead to the increase in secondary metabolite production, which is likely to indirectly occur by the increase in nutrient uptake [38].

The novel Glomeromycota taxa that were found in our study confirmed the observed high diversity of the AM in herb-rich coniferous forests [31]. As is often the case in metagenomic studies, especially in rich metagenomes such as soil, we found several new genera within Glomeromycota, which at this point are difficult to taxonomically describe with precision because they are unculturable under laboratory conditions.

Thus, speaking of the potential application of our results in agriculture, we can consider arbuscular mycorrhiza as an enhancer of nutrient availability for host plants. From this we can conclude that conventional farmer fertilization practices (FFP) based on nutrient addition can be optimized with respect to their effect on mycorrhizal fungi, thereby increasing the richness and diversity of fungal communities, and through them influencing soil productivity. These findings are consistent with those of Muneer et al. [39], who compared the effects of conventional FFP and different nutrient management practices on fungal composition and diversity in red soils.

Of course, properties of such complex ecosystems cannot be attributed to specific fungal components alone. Both fungi and bacteria play a role in the active degradation of plant biomass, by encoding a distinct pool of carbohydrate-active enzymes (CAZymes—glycoside hydrolases, glycosyltransferases, etc.) and demonstrating the interplay in the maintenance of the ecosystem [40]. Preliminary results of the bacterial diversity of the Chernevaya taiga soils revealed specific microbial components of this ecosystem [1,32]. The combination of the results of the bacterial and fungal community analysis can provide a better understanding of the Chernevaya taiga soil properties, such as giant plant growth, and may provide us with novel tools for soil productivity improvement.

## Figures and Tables

**Table 1:** Soil sampling locations, topsoil layer (soil properties are described in detail in [1]).

Sample ID	Description	Coordinates
T1	Chernevaya taiga, Kemerovo oblast, 38 km of railway Tomsk—Taiga station. Fir stands with an admixture of aspen.	56.18399 85.28246
T3	Control point, Tomsk oblast, block 86, 1 km left of Shigarsky tract. Aeolian-fluvial plain underlain by loamy deposits, poorer community—birch-aspen-pine forest, fern-broadgrass.	56.28826 84.48016

**Table 2:** Differential abundance fungal taxa in Tomsk region. Negative log2FC values correspond to the taxa that are more abundant in Chernevaya taiga samples. Threshold for the log2FC was set at 2.0 and the FDR-adjusted *p*-value cutoff at 0.05.

**Tomsk T1-T3 Spring**					
#	baseMean	log2FC	lfcSE	Rank1	Rank2
1	477.04	−11.47	1.17	k__Fungi	p__Glomeromycota
2	60.64	5.81	1.12	k__Fungi	p__Calcarisporiellomycota
**Tomsk T1-T3 Summer**					
#	baseMean	log2FC	lfcSE	Rank1	Rank2
1	29339.96	2.59	0.47	k__Fungi	p__Basidiomycota
2	636.93	4.00	1.24	k__Fungi	p__Mucoromycota
3	109.29	−3.28	0.49	k__Fungi	p__Rozellomycota
**Tomsk T1-T3 Fall**					
#	baseMean	log2FC	lfcSE	Rank1	Rank2
1	81.89	2.98	0.66	k__Fungi	p__unidentified
2	107.28	3.80	0.80	k__Fungi	p__Mucoromycota
3	13544.93	−2.44	0.39	k__Fungi	p__Mortierellomycota
4	42.61	−3.39	0.74	k__Fungi	p__Rozellomycota
5	100.61	−4.11	1.56	k__Fungi	p__Glomeromycota

**Table 3:** Novel genera of the Glomeromycota in the studied samples, differentially abundant between samples. Log2FC < 0 means that taxa is more abundant in Chernevaya taiga soils than in control, and vice versa.

baseMean	log2FC	padj	Kingdom	Family	Genus	Species	Trophic Mode	Guild
14.72	7.21	0.03	Fungi	Glomeraceae	unidentified	unidentified	Symbiotroph	Arbuscular mycorrhizal
48.45	−9.15	0.00	Fungi	Claroideoglomeraceae	unidentified	unidentified	Symbiotroph	Arbuscular mycorrhizal
26.54	−8.28	0.00	Fungi	Claroideoglomeraceae	unidentified	unidentified	Symbiotroph	Arbuscular mycorrhizal
26.18	−8.27	0.00	Fungi	Claroideoglomeraceae	unidentified	unidentified	Symbiotroph	Arbuscular mycorrhizal
13.77	−7.34	0.00	Fungi	Glomeraceae	Glomus	unidentified	Symbiotroph	Arbuscular mycorrhizal

## Data Availability

The data presented in this study are openly available in Figshare at https://doi.org/10.6084/m9.figshare.16565721.v3 (accessed on 12 September 2021).

## Supplementary Materials

The following are available online at https://www.mdpi.com/article/10.3390/jof7110908/s1: Figure S1: Venn diagrams showing the number of distinct or common ASVs detected in the fungal communities studied in this work. Top left: Chernevaya taiga samples, different seasons. Top right: control samples, different seasons. Bottom: comparison of Chernevaya taiga and control samples (all ASVs from different seasons combined).
