# Supplementary material for: Fungal Metagenome of Chernevaya Taiga Soils: Taxonomic Composition, Differential Abundance and Factors Related to Plant Gigantism
Source: J Fungi (Basel). 2021 Oct 27;7(11):908. doi: 10.3390/jof7110908 (PMC8620766; doi:10.3390/jof7110908)
Supplement: Supplementary file 1 [file jof-07-00908-s001.zip › jof-1398401-supplementary.pdf]

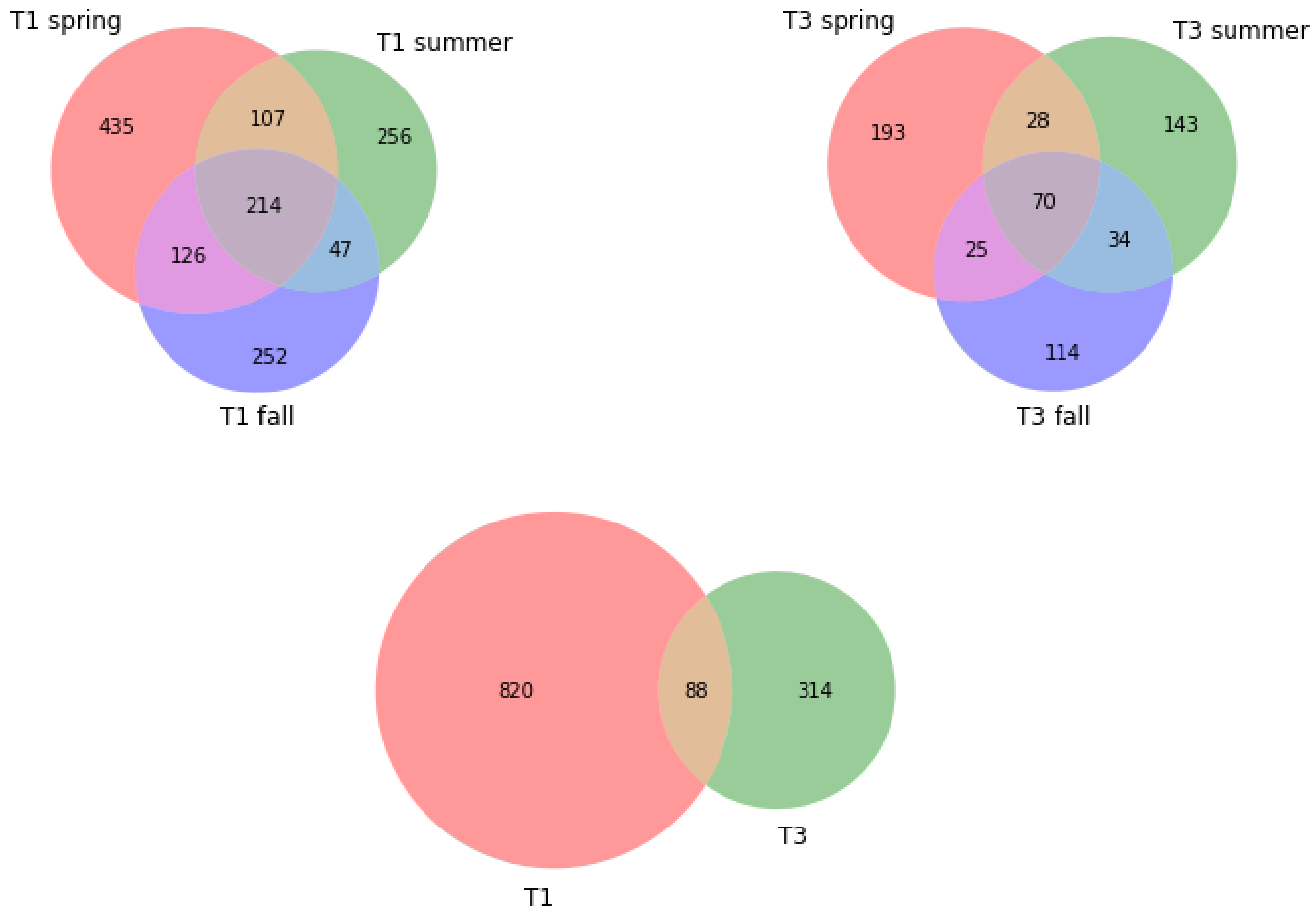

Figure S1. Venn diagrams showing the number of distinct or common ASVs detected in the fungal communities studied in this work. Top left: Chernevaya taiga samples, different seasons. Top right: control samples, different seasons. Bottom: comparison of Chernevaya taiga and control samples (all ASVs from different seasons combined).
